# Cancer burden attributable to risk factors, 1990–2019: A comparative risk assessment

**DOI:** 10.1016/j.isci.2024.109430

**Published:** 2024-03-08

**Authors:** Junjie Hu, Hongliang Dong, Yiming Dong, Runxuan Zhou, Wilhem Teixeira, Xingxing He, Da-Wei Ye, Gang Ti

**Affiliations:** 1 Cancer Center, Tongji Hospital, Tongji Medical College, Huazhong University of Science and Technology, Wuhan 430030, China; 2 Department of GI Surgery, Tongji Hospital, Tongji Medical College, Huazhong University of Science and Technology, Wuhan 430030, P.R. China; 3 GI Cancer Research Institute, Tongji Hospital, Tongji Medical College, Huazhong University of Science and Technology, Wuhan 430030, P.R. China; 4 Department of Orthopedics, Tongji Hospital, Tongji Medical College, Huazhong University of Science and Technology, Wuhan 430030, China; 5 Tongji Medical College, Huazhong University of Science and Technology, Wuhan 430030, China; 6 Department of Gastroenterology, Zhongnan Hospital of Wuhan University, Wuhan 430030, China; 7 Hubei Clinical Center and Key Laboratory of Intestinal and Colorectal Diseases, Wuhan 430030, China; 8 Department of Medical Record, Shanxi Bethune Hospital, Shanxi Academy of Medical Sciences, Tongji Shanxi Hospital, Third Hospital of Shanxi Medical University, Taiyuan 030032 Shanxi, China

**Keywords:** Cancer, Health sciences, Public health

## Abstract

An up-to-date comprehensive assessment of the cancer burden attributable to risk factors is essential for cancer prevention. We analyzed the population attributable fraction (PAF) of cancer disability-adjusted life years (DALYs) attributable to 11 level 2 risk factors using data from the Global Burden and Disease Study (GBD) 2019. We highlighted that almost half of the cancer DALYs can be preventable by modifying relevant risk factors. The attributable cancer DALYs increased by 60.42%–105.0 million from 1990 to 2019. Tobacco, dietary risks, alcohol use, high body-mass index, and air pollution were the top five risk factors. The PAFs attributable to high fasting plasma glucose, high body-mass index, and low physical activity have increased worldwide from 1990 to 2019. Unsafe sex was the leading risk factor for women before age of 54. Tailored prevention programs targeted at specific populations should be scaled up to reduce the cancer burden in the future.

## Highlights

• Nearly half of cancer burden could be preventable
• Tobacco, dietary risks, alcohol use, high BMI, and air pollution were the major risks
• The PAFs of high FPG, high BMI and low physical activity increased worldwide
• Unsafe sex was the leading risk factor in females before 54 years old

## Graphical abstract

**Figure:**
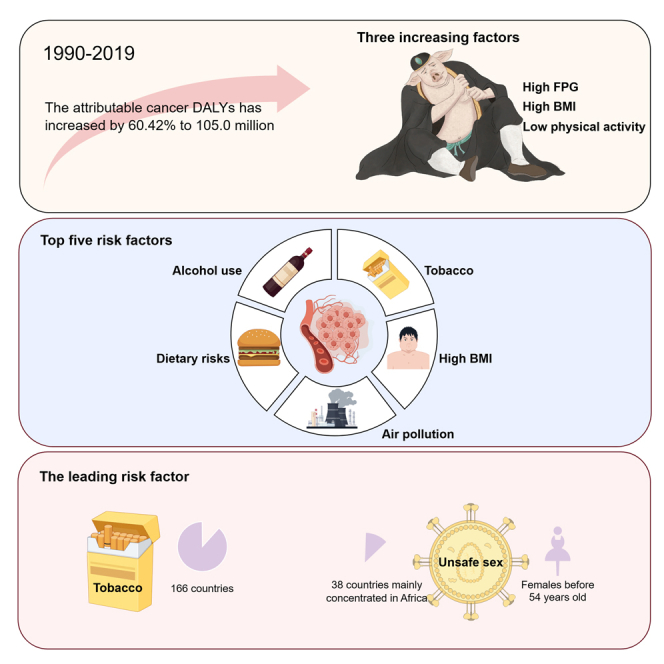

## Introduction

Cancer remains a major threat to global human health worldwide. Among 22 groups of diseases and injuries, cancer was reported as the second leading cause of death and disability-adjusted life years (DALYs) after cardiovascular disease.^1^ It was estimated that cancer was responsible for 23.6 million new cases, 10.0 million deaths, and 250 million DALYs globally in 2019, with increases in cancer incidence, mortality and DALYs of 26.3%, 20.9%, and 16.0%, respectively, from 2010 to 2019.^2^ In addition, the World Health Organization predicted that global cancer incidence will increase to 24 million by 2035.^3^ Primary prevention, early diagnosis and early treatment are the three major weapons against cancer. However, due to the limited medical resources and the high cost of therapies, people in many countries, including subpopulations in very high-income countries, have no access to comprehensive and systematic curative treatment for cancer. To date, therapeutic interventions have not significantly reduced overall cancer mortality, although some progress has been made for specific cancers.^4^ In this case, primary prevention has been identified as the optimal choice for reducing the global cancer burden. As reported by the World Cancer Research Fund, about one-third to one-half of all cancers may be preventable by modifying known environmental, lifestyle, and infection-related risk factors.^5^ Nevertheless, low budgets have been allocated to cancer prevention efforts even in Europe and the United States, due to the long cycle of cancer development, which requires time to see the benefits.^6^

Previous epidemiological studies have reported the cancer burden attributable to potentially modifiable risk factors. However, most of these studies were restricted to earlier years, specific regions or countries, and limited sample sizes.^7^^,^^8^^,^^9^^,^^10^^,^^11^ In addition, most studies defined cancer burden mainly as morbidity or mortality, ignoring the additional health burden such as living with disability, making it difficult for policymakers to prioritize resource allocation. Besides, most of previous Global Burden and Disease Study (GBD) studies tended to focus on the cancer burden attributable to a specific risk factor, such as high fasting plasma glucose (FPG), tobacco, excess body weight, and alcohol use, without further comparative assessment of all risk factors.^12^^,^^13^^,^^14^^,^^15^ A recent GBD study led by the GBD 2019 Cancer Risk Factors Collaborators reported the cancer mortality and DALYs attributable to potentially modifiable risk factors. Although there were some references to more detailed risk factors, most of this study focused on the cancer burden attributable to level 1 risk factors.

In this study, we focused on the population attributable fraction (PAF) of cancer DALYs attributable to 11 level 2 risk factors, and highlighted the disparities in sex, age, SDI, locations, and cancer types. We hope that our study can raise policymakers’ concerns about cancer prevention and help them prioritize cancer prevention agendas in the future.

## Results

### Global attributable burden

**Figure 1:**
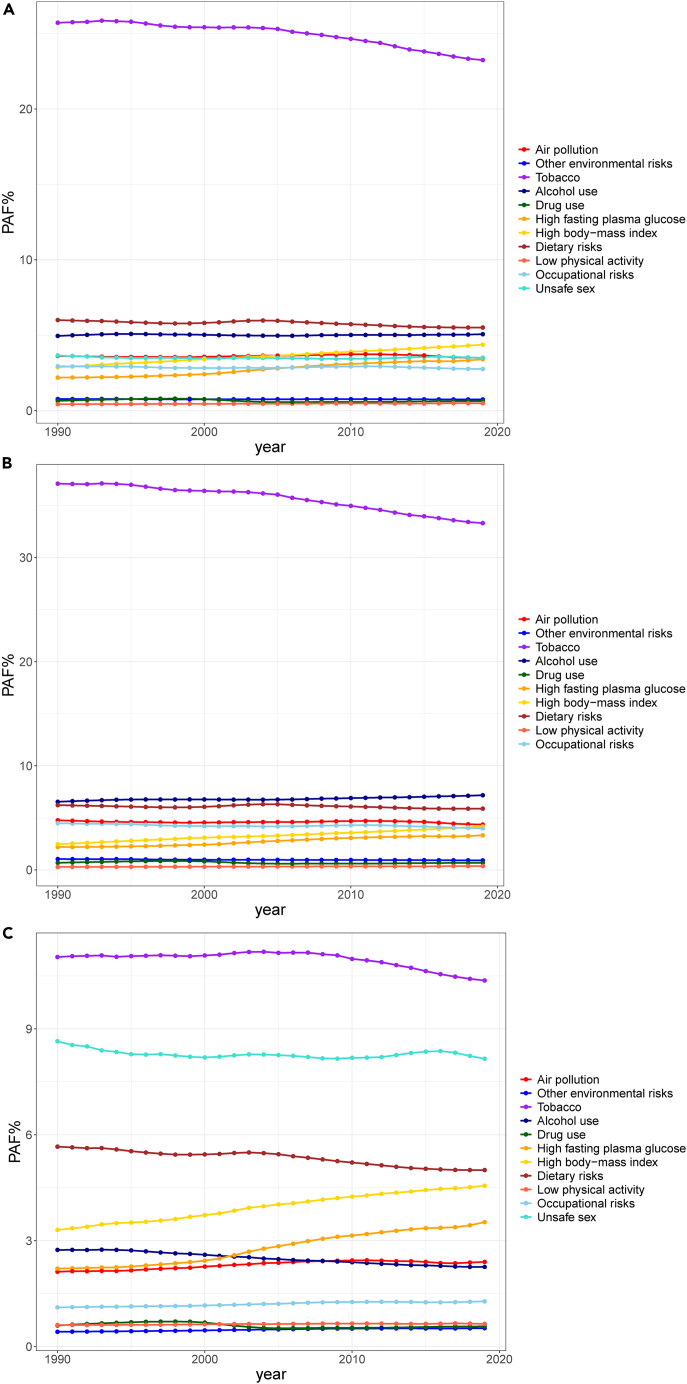
PAFs of cancer DALYs attributable to eleven level 2 risk factors at the global level The temporal trends of global age-standardized PAFs of cancer DALYs attributable to eleven level 2 risk factors for both sexes (A), male (B) and female (C) from1990 to 2019. DALYs, disability-adjusted life years. PAF, population attributable fraction. See also Figures S1 and S2.

In 2019, there were 105.0 (95%UI, 95.0 to 116.3) million cancer DALYs or 41.23% (95%UI, 38.39%–44.83%) of total cancer DALYs attributable to all modifiable risk factors worldwide, with 67.5 (95%UI,60.8 to 75.1) million male cancer DALYs or 47.22% (95%UI, 44.59%–50.62%) of all male cancer DALYs and 37.6 million (95%UI, 32.8 to 43.1) female cancer DALYs or 33.55% (95%UI, 30.25%–37.79%) of all female cancer DALYs attributable to all modifiable risk factors. The top five level 2 risk factors for attributable cancer DALYs were tobacco, dietary risks, alcohol use, high body-mass index (BMI), and air pollution, accounting for 23.24% (95%UI, 22%–24.5%), 5.51% (95%UI, 4.19%–7.47%), 5.07% (95%UI, 4.57%–5.57%), 4.37% (95%UI, 2.52%–6.73%), and 3.5% (95%UI, 2.65%–4.36%) of cancer DALYs, respectively. The top five level 2 risk factors for attributable cancer DALYs in men were tobacco, alcohol use, dietary risks, air pollution, and occupational risks. In contrast, tobacco, unsafe sex, dietary risks, high body-mass index (BMI), and high FPG were the top five level 2 risk factors for women. High BMI, high FPG, and low physical activity contributed more to cancer DALYs in women than in men. Unsafe sex was a female-specific risk factor. The other seven level 2 risk factors contributed more to cancer DALYs in men than in women (Table 1; Figure 1).

**Table 1:** Global estimates of number, rate, and PAF of cancer DALYs attributable to eleven level 2 risk factors and the percent change by sex from 1990 to 2019

Risk factors	Sex	DALYs			Age-standardized DALYs rate			Age-standardized PAF%		
		DALYs in 1990 (×1000) (95% UI)	DALYs in 2019 (×1000) (95% UI)	Percent change,1990–2019 (95% UI)	Age-standardized DALYs rate per 100,000 (95% UI) in 1990	Age-standardized DALYs rate per 100,000 (95% UI) in 2019	Percent change, 1990–2019 (95% UI)	Age-standardized PAF%, 1990 (95% UI)	Age-standardized PAF%, 2019 (95% UI)	Percent change,1990–2019 (95% UI)
All risk factors	Male	42898.31 (39456.67–46816.72)	67474 (60781.7–75100.25)	57.29 (40.99–76.05)	2226.75 (2053.09–2425.56)	1711.58 (1546.87–1903.48)	−23.14 (−30.88–14.08)	48.39 (45.75–51.92)	47.22 (44.59–50.62)	−2.41 (−5.49–1.17)
	Female	22580.18 (20284.89–25627.41)	37563.38 (32771.12–43142)	66.36 (52.49–80.67)	1037.14 (931.11–1179.24)	866.87 (756.6–994.88)	−16.42 (−23.22–9.32)	32.62 (29.69–36.72)	33.55 (30.25–37.79)	2.85 (−1.4–7.22)
	Both	65478.5 (60651.09–71834.86)	105037.38 (95022.5–116316.26)	60.42 (48.46–73.93)	1587.05 (1470.08–1739.1)	1262.68 (1142.77–1398.69)	−20.44 (−26.27–13.95)	41.51 (38.96–45.27)	41.23 (38.39–44.83)	−0.66 (−3.86–2.92)
Air pollution	Male	4291.25 (3206.09–5475.36)	6253.19 (4636.88–8073.24)	45.72 (16.91–79.34)	219.38 (164.43–280.04)	157.86 (116.76–203.64)	−28.04 (−42.13–11.69)	4.76 (3.59–5.98)	4.35 (3.28–5.42)	−8.64 (−22.55–6.22)
	Female	1463.54 (1081.45–1881.43)	2698.78 (1998.45–3392.94)	84.4 (49.38–123.88)	67.37 (49.8–86.71)	61.97 (45.9–77.89)	−8.02 (−25.41–11.57)	2.12 (1.6–2.69)	2.4 (1.81–2.98)	13.16 (−5.71–30.96)
	Both	5754.8 (4305.32–7280.69)	8951.97 (6680.89–11342.6)	55.56 (27.68–84.64)	138.76 (103.77–175.57)	107.36 (80.13–136.04)	−22.63 (−36.4–8.22)	3.63 (2.74–4.55)	3.5 (2.65–4.36)	−3.39 (−17.3–11.12)
Other environmental risks	Male	925.51 (180.03–1773.67)	1299.59 (257.51–2535)	40.42 (24.22–60.66)	47.76 (9.31–91.42)	32.99 (6.55–64.39)	−30.92 (−38.8–20.96)	1.04 (0.2–1.97)	0.91 (0.18–1.76)	−12.29 (−19.67–4.74)
	Female	287.99 (56.43–558.2)	585.89 (114.71–1135.51)	103.44 (82.86–126.8)	13.29 (2.6–25.78)	13.44 (2.63–26.04)	1.14 (−9.1–12.72)	0.42 (0.08–0.81)	0.52 (0.1–1.01)	24.53 (15.39–34.66)
	Both	1213.5 (236.75–2337.37)	1885.48 (373.71–3652.49)	55.37 (39.87–73.68)	29.43 (5.75–56.7)	22.66 (4.49–43.94)	−22.98 (−30.62–13.94)	0.77 (0.15–1.48)	0.74 (0.15–1.43)	−3.8 (−10.86–3.83)
Tobacco	Male	32710 (30454.61–35030.14)	47632.1 (42934.91–52715.37)	45.62 (29.94–63.06)	1707.61 (1595.13–1826.31)	1207.72 (1087.44–1334.92)	−29.27 (−36.68–20.84)	37.11 (35.43–38.71)	33.31 (31.78–34.85)	−10.22 (−13.42–6.44)
	Female	7552.81 (6921.15–8229.9)	11691.47 (10574.83–12864.46)	54.8 (45.09–66.15)	350.69 (321.29–381.94)	267.84 (242.1–294.85)	−23.62 (−28.42–18.03)	11.03 (10.11–12.01)	10.37 (9.61–11.18)	−6.02 (−10.96–0.71)
	Both	40262.81 (37733.77–42824.82)	59323.57 (54003.69–64819.13)	47.34 (34.02–62.05)	983.22 (921.4–1044.24)	711.71 (648.94–777.11)	−27.61 (−34.01–20.43)	25.71 (24.48–26.89)	23.24 (22–24.5)	−9.63 (−14.16–4.04)
Alcohol use	Male	6073.94 (5373.48–6859.18)	10470.27 (9178.12–11794.88)	72.38 (50.69–96.19)	300.94 (266.87–339.62)	259.87 (227.84–292.93)	−13.65 (−24.25–2.22)	6.54 (5.83–7.24)	7.17 (6.46–7.88)	9.65 (2.16–18.37)
	Female	1885.77 (1662.24–2115.74)	2521.1 (2221.87–2852.97)	33.69 (24.73–43.63)	86.99 (76.55–97.6)	58.25 (51.39–65.9)	−33.04 (−37.52–27.94)	2.74 (2.41–3.05)	2.26 (1.99–2.51)	−17.59 (−21.89–13.01)
	Both	7959.7 (7156.1–8853.33)	12991.38 (11579.92–14521.47)	63.21 (45.93–80.88)	189.37 (170.39–210.22)	155.19 (138.4–173.5)	−18.05 (−26.46–9.2)	4.95 (4.46–5.45)	5.07 (4.57–5.57)	2.31 (−4.94–10.67)
Drug use	Male	624.56 (498.87–767.59)	966.06 (783.59–1178.98)	54.68 (30.03–85.11)	31.29 (25.02–38.38)	24.52 (20.03–29.93)	−21.66 (−34.56–6.21)	0.68 (0.55–0.83)	0.68 (0.56–0.81)	−0.51 (−13.26–14.51)
	Female	411.46 (267.85–585.83)	645.12 (491.4–834.94)	56.79 (21.31–107.09)	18.97 (12.33–26.95)	14.76 (11.25–19.09)	−22.19 (−39.73–2.83)	0.6 (0.39–0.83)	0.57 (0.44–0.72)	−4.16 (−22.9–21.81)
	Both	1036.02 (785.85–1329.89)	1611.18 (1294.86–1992.7)	55.52 (32.44–85.6)	24.82 (18.71–31.72)	19.36 (15.58–23.94)	−21.98 (−33.6–6.99)	0.65 (0.5–0.83)	0.63 (0.51–0.77)	−2.56 (−15.5–12.79)
High fasting plasma glucose	Male	1798.2 (415.55–3958.75)	4599.74 (1118.15–9896.34)	155.8 (132.17–191.59)	100.9 (23.56–220.05)	120.41 (29.51–257.67)	19.35 (8.68–35.12)	2.19 (0.51–4.84)	3.32 (0.81–7.08)	51.57 (44.06–66.1)
	Female	1489.43 (389.54–3114.13)	3981.04 (1085.16–8399.12)	167.29 (150.1–191.33)	70.24 (18.4–146.8)	90.98 (24.82–192.11)	29.54 (20.92–40.89)	2.21 (0.58–4.6)	3.52 (0.95–7.28)	59.51 (51.4–70.65)
	Both	3287.64 (846.22–6862.88)	8580.78 (2357.08–17568.24)	161 (143.65–185.3)	83.74 (21.78–174.19)	104.24 (28.65–212.87)	24.49 (16.4–35.63)	2.19 (0.57–4.52)	3.41 (0.92–6.89)	55.51 (49.05–66.61)
High body-mass index	Male	2236.51 (927.57–4178.39)	6011.22 (3091.71–9904.24)	168.78 (118.85–249.4)	113.66 (47.36–211.1)	150.73 (77.13–247.49)	32.62 (8.99–71.3)	2.47 (1.02–4.62)	4.16 (2.13–6.81)	68.44 (43.42–113.68)
	Female	2249.66 (1220.71–3653.5)	5164.09 (3126.66–7686.54)	129.55 (101.31–169.46)	105.02 (57.06–170.49)	117.76 (71.34–175)	12.13 (−1.5–31.31)	3.3 (1.78–5.33)	4.56 (2.74–6.79)	37.9 (22.53–60.77)
	Both	4486.18 (2204.38–7620.31)	11175.31 (6355.93–17263.74)	149.11 (113.39–201.15)	109.89 (54.34–186.18)	133.93 (76.19–206.81)	21.88 (5.09–46.48)	2.87 (1.43–4.88)	4.37 (2.52–6.73)	52.16 (32.96–82.08)
Dietary risks	Male	5491.49 (3804.63–8415.83)	8347.44 (6061.8–11645.55)	52.01 (28.61–80.91)	286.43 (199.3–434.54)	213.16 (154.96–296.5)	−25.58 (−36.57–11.91)	6.22 (4.4–9.41)	5.88 (4.37–8.16)	−5.5 (−16.15–6.69)
	Female	3877.51 (2947.91–5549.63)	5603.85 (4280.45–7319.49)	44.52 (25.89–64.44)	180.01 (137.19–257.26)	129.18 (98.68–168.67)	−28.24 (−37.27–18.76)	5.66 (4.28–8.03)	5 (3.9–6.54)	−11.71 (−20.97–2.66)
	Both	9369 (6792.06–13895.47)	13951.29 (10502.65–18791.39)	48.91 (28.62–69.7)	229.56 (167.29–339.02)	168.77 (127.14–226.89)	−26.48 (−36.14–16.38)	6 (4.39–8.86)	5.51 (4.19–7.47)	−8.21 (−18.13–2.21)
Low physical activity	Male	205.58 (42.4–427.32)	478.9 (111.52–952.31)	132.95 (108.55–174.59)	12.89 (2.69–26.41)	13.26 (3.12–26.37)	2.91 (−6.94–19.01)	0.28 (0.06–0.57)	0.37 (0.09–0.72)	30.66 (17.58–53.42)
	Female	402.34 (185.02–688.84)	723.76 (337.94–1209.09)	79.89 (65.52–99.48)	19.34 (8.87–33.15)	16.58 (7.77–27.65)	−14.28 (−20.65–5.21)	0.61 (0.27–1.03)	0.64 (0.31–1.06)	5.46 (−1.78–15.13)
	Both	607.92 (231.75–1111.35)	1202.65 (454.74–2164.86)	97.83 (82.79–118.02)	16.35 (6.17–29.88)	14.98 (5.67–26.9)	−8.37 (−14.84–0.35)	0.43 (0.16–0.77)	0.49 (0.19–0.88)	14.37 (6.37–25.81)
Occupational risks	Male	3760.3 (2900.77–4643.57)	5523.05 (4272.77–6829.14)	46.88 (34.91–60.08)	206.29 (159.87–254.45)	144.38 (111.96–178.45)	−30.01 (−35.34–23.8)	4.49 (3.46–5.58)	3.98 (3.13–4.87)	−11.17 (−16.71–5.55)
	Female	765.15 (568.94–960.95)	1441.73 (1067.08–1829.69)	88.42 (59.76–119.65)	35.24 (26.15–44.36)	33.1 (24.5–42.01)	−6.06 (−20.57–9.51)	1.11 (0.82–1.38)	1.28 (0.98–1.58)	15.57 (1.47–29.57)
	Both	4525.45 (3573–5519.3)	6964.78 (5467.88–8580.43)	53.9 (42.46–66.72)	112.44 (88.83–136.89)	84.42 (66.24–103.71)	−24.92 (−30.39–18.78)	2.94 (2.32–3.58)	2.76 (2.19–3.34)	−6.28 (−11.05–1.13)
Unsafe sex	Female	6176.25 (5437.67–7316.93)	8955.01 (7547.73–9978.46)	44.99 (22.92–67.74)	275.05 (242.75–326.15)	210.64 (177.67–234.85)	−23.42 (−35.17–11.64)	8.65 (7.79–10.06)	8.15 (6.97–8.78)	−5.73 (−17.64–5.19)
	Both	6176.25 (5437.67–7316.93)	8955.01 (7547.73–9978.46)	44.99 (22.92–67.74)	139.98 (123.79–165.85)	107.2 (90.52–119.43)	−23.42 (−35.05–11.78)	3.66 (3.28–4.28)	3.5 (2.98–3.85)	−4.36 (−18.72–8.33)

DALYs, disability-adjusted life years. PAF, population attributable fraction.

Although the absolute number of DALYs has increased by 60.42% (95%UI, 48.46%–73.93%) from 1990 to 2019, the age-standardized DALYs rate (ASDR) attributable to combined modifiable risk factors decreased from 1587.05 (95%UI, 1470.08 to 1739.1) to 1262.68 (95%UI, 1142.77 to 1398.69) per 100 000 population. Similar trends were observed for most level 2 risk factors, except for high FPG and high BMI, whose attributable ASDR increased from 1990 to 2019 (Table 1; Figures S1 and S2). The PAF attributable to combined modifiable risk factors remained relatively stable during the observed period (−0.66; 95%UI, −3.86 to 2.92). To be specific, the PAF attributable to tobacco, drug use, and dietary risks decreased, while the PAF attributable to high FPG, high BMI, and low physical activity increased in both men and women. In addition, the PAF attributable to air pollution, other environmental risks and occupational risks increased in women but decreased in men, whereas the PAF attributable to alcohol use increased in men but decreased in women. Downward trends in the PAF attributable to unsafe sex were observed for females (Table 1; Figure 1).

### Regional distribution of attributable burden

**Figure 2:**
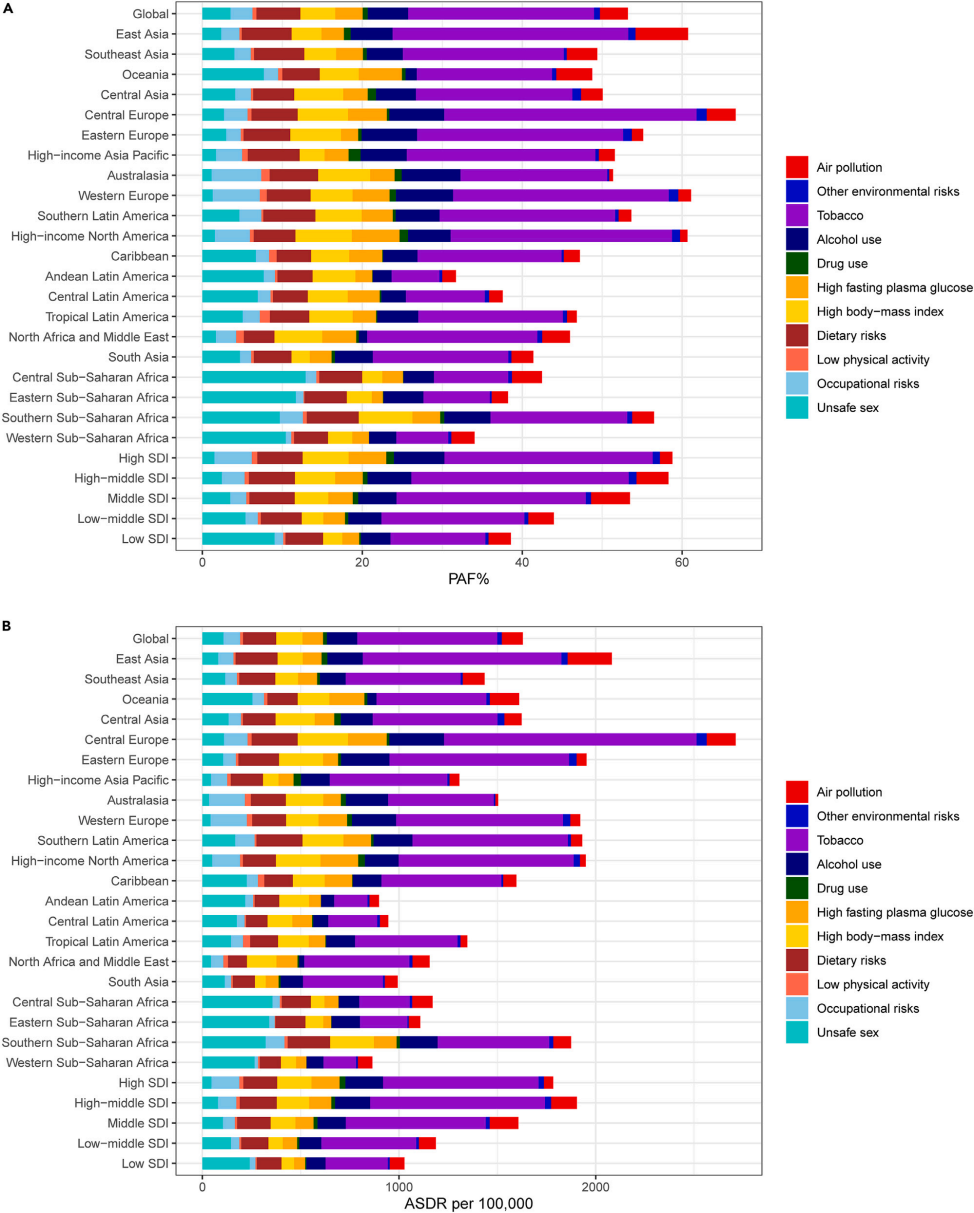
PAFs and rates of cancer DALYs attributable to eleven level 2 risk factors at the regional level (A) Age-standardized PAFs of cancer DALYs attributable to eleven level 2 risk factors, for both sexes in 27 global and regional locations in 2019. (B) Age-standardized DALY rates of cancer attributable to eleven level 2 risk factors, for both sexes in 27 global and regional locations in 2019. DALYs, disability-adjusted life years. PAF, population attributable fraction. See also Table S1.

The PAF attributable to 11 level 2 risk factors varied considerably across regions (Table S1; Figure 2). The highest PAF for combined risk factors was observed in Central Europe (49.41%; 95%UI, 46.48%–52.86%). For specific risk factors, the highest PAF for air pollution was observed in East Asia (6.58%; 95%UI, 4.98%–8.05%), for other environmental risks in Central Europe (1.26%; 95%UI, 0.22%–2.45%), for tobacco in Central Europe (31.56%; 95%UI, 30.3%–32.91%), for alcohol use in Australasia (7.36%; 95%UI, 6.58%–8.14%), for drug use in high-income Asia Pacific (1.49%; 95%UI, 1.04%–2.09%), for high FPG in high-income North America (5.95%; 95%UI, 1.69%–11.78%), for high BMI in high-income North America (7.06%; 95UI, 4.47%–9.64%), for dietary risks in Southern Latin America (6.52%; 95%UI, 5.04%–8.23%), for low physical activity in Tropical Latin America (1.27%; 95%UI, 0.61%–2%), for occupational risks in Australasia (6.2%; 95%UI, 5.13%–7.27%), and for unsafe sex in Central sub-Saharan Africa (12.9%; 95%UI, 10.08%–15.8%). Notably, tobacco and dietary risks index were the common top five risk factors across all 21 GBD regions (Table S1; Figure 2A).

ASDR showed comparable distribution patterns to PAF (Table S1; Figure 2B).

### National attributable burden by SDI

**Figure 3:**
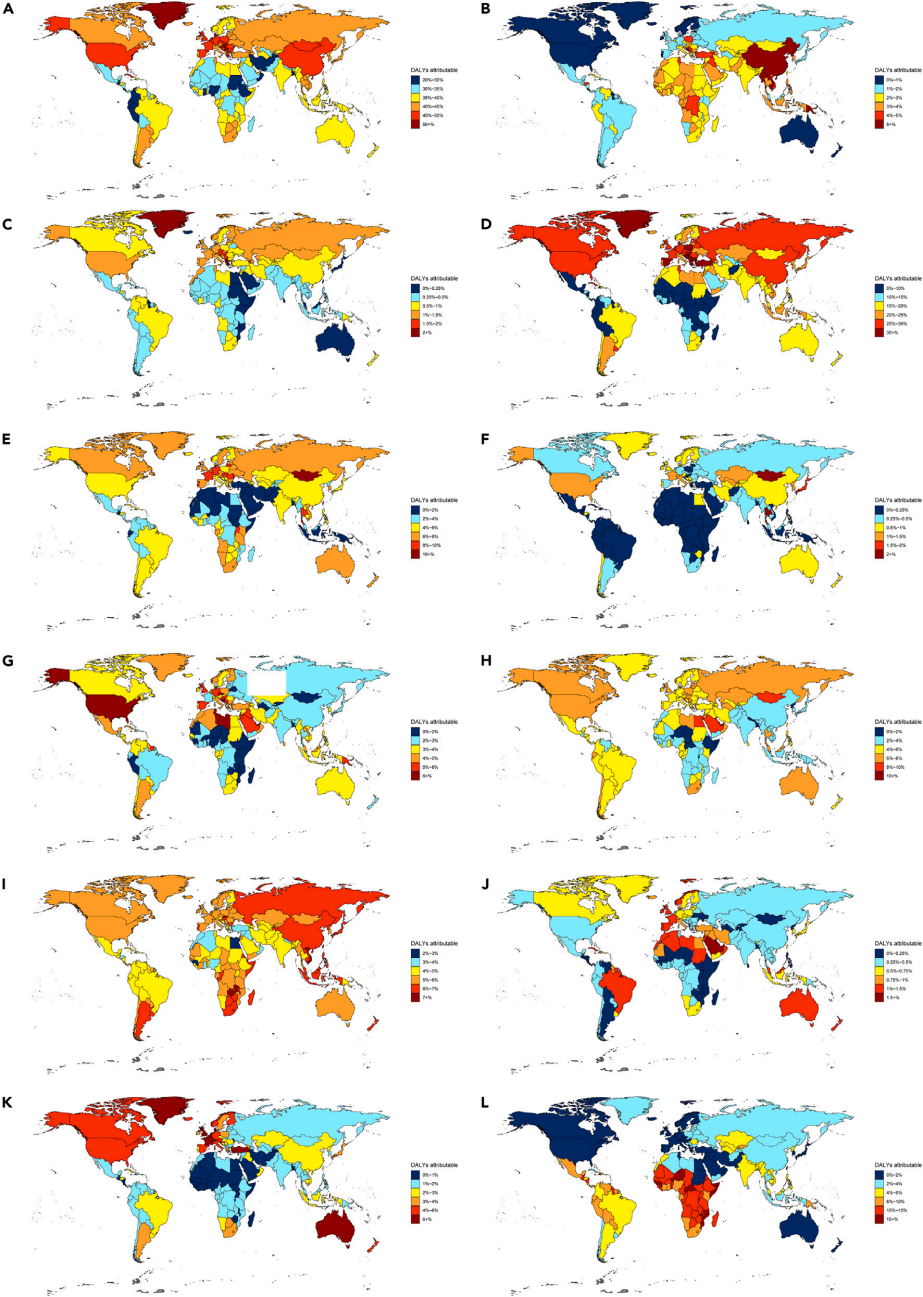
PAFs of cancer DALYs attributable to eleven level 2 risk factors at the national level Age-standardized PAF of cancer DALYs attributable to all modifiable risk factors (A) and eleven specific level 2 risk factors: air pollution (B), other environmental risks (C), tobacco (D), alcohol use (E), drug use (F), high fasting plasma glucose (G), high body-mass index (H), dietary risks (I), low physical activity (J), occupational risks (K), unsafe sex (L), for both sexes in 204 countries and territories in 2019. DALYs, disability-adjusted life years. PAF, population attributable fraction. See also Table S2 and Figure S3.

The PAF also varied considerably among countries (Table S2; Figure 3). In 2019, the PAF of cancer DALYs (more than 40%) attributable to combined modifiable risk factors was higher in more developed locations, mainly in North America, Asia, and Europe. In contrast, the PAF was lower (less than 40%) in less developed areas, mainly in Africa, South America, and Oceania. Alarmingly, the PAF exceeded 50% in Serbia, Hungary, Kiribati, Montenegro, and Greenland. To be specific, the highest PAF for air pollution, other environmental risks, tobacco, alcohol use, drug use, high FPG, high BMI, dietary risks, low physical activity, occupational risks, and unsafe sex was observed in China (6.66%; 95%UI, 5.05%–8.13%), Albania (2.34%; 95%UI, 0.42%–5%), Montenegro (40.4%; 95%UI, 38.31%–42.43%), Mongolia (14.46%; 95%UI, 11.26%–18.06%), Mongolia (3.48%; 95%UI, 1.89%–5.94%), Bahrain (10.64%; 95%UI, 3.47%–19.34%), Qatar (11.02%; 95%UI, 7.29%–14.74%), Zambia (7.9%; 95%UI, 6.12%–9.64%), Saudi Arabia (2.05%; 95%UI, 1.12%–3.13%), Netherlands (7.73%; 95%UI, 6.25%–9.21%), and Kiribati (22.85%; 95%UI, 20%–26.76%), respectively. Notably, tobacco was the leading risk factor in 166 countries, whereas unsafe sex was the leading risk factor in the remaining 38 countries, mainly concentrated in Africa (Table S2).

Figure S3 shows the correlations between the PAF attributable to selected risk factors and SDI in 204 countries and territories in 2019. The PAFs for other environmental risks, tobacco, alcohol use, drug use, high FPG, high BMI, low physical activity, and occupational risks were positively correlated with SDI, whereas the PAFs for air pollution and unsafe sex were negatively correlated with SDI. No significant correlation was observed between dietary risks and SDI.

### Attributable burden by age group and sex

**Figure 4:**
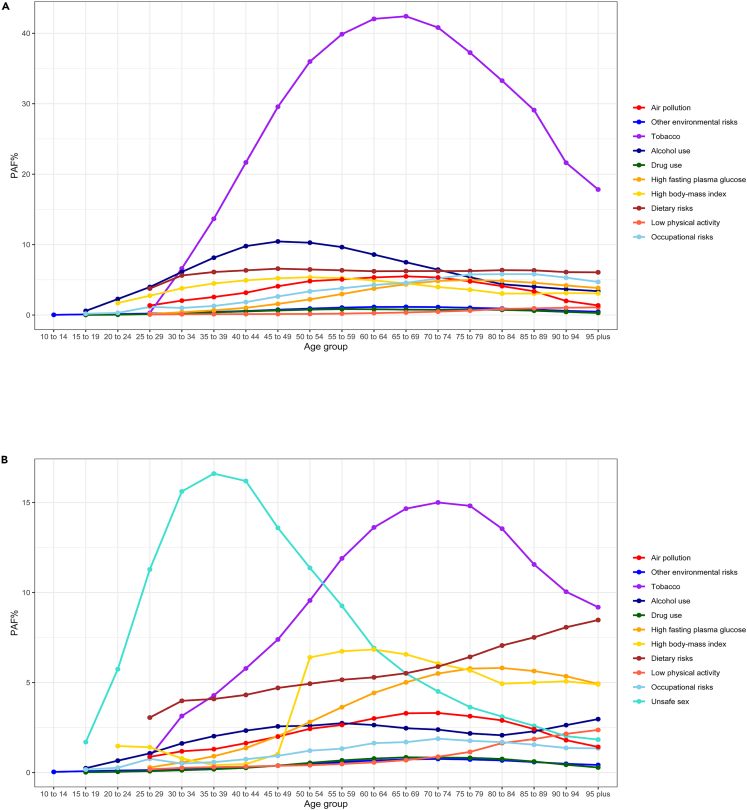
Age and sex disparity in the PAFs of cancer DALYs attributable to eleven level 2 risk factors at the global level Global age-specific PAF of cancer DALYs attributable to eleven specific level 2 risk factors for male(A) and female(B) in 2019. DALYs, disability-adjusted life years. PAF, population attributable fraction. See also Table S1.

In general, the age-specific PAF followed a unimodal distribution for most level 2 risk factors, with the highest PAF observed in middle-aged and older individuals. However, the PAFs for dietary risks and low physical activity increased or remained stable with increasing age in both men and women. (Figure 4). The PAF for air pollution peaked in the 65–69 age group for both men and women, with men having higher PAFs than women. The highest PAFs for both other environmental risk factors and tobacco were observed in the 65–69 age group for men and in the 70–74 age group for women, with men higher than women. The PAF for alcohol use peaked in the 45–49 age group for men and in the 50–59 age group for women. For drug use, the PAF was highest in the 55–59 age group for men and in the 65–69 age group for women, with men being higher up to the 60–64 age group and women reversing this trend from the 65–69 age group onwards. For high FPG, the highest PAF was observed in the 75–79 age group for men and in the 80–84 age group for women, with women having consistently higher PAF except in the 50–54 age group. For high BMI, the PAF was highest in men aged 50–54 years and in women aged 60–64 years. The PAF was significantly higher in men in the early age, while this pattern was reversed from the 50–54 age group onwards, as a steep increase was observed in women in this age group. For dietary risks, men had a higher PAF than women in the early age groups, whereas women’s PAF exceeded that of men from the age group 75–79. For low physical activity, women had a higher PAF than men in all age groups. For occupational risks, men aged 85–89 years had the highest PAF, while women aged 70–74 years had the highest PAF, with men being consistently higher than women. The PAF attributable to unsafe sex reached a plateau in women aged 35–39 years. Notably, unsafe sex surpassed tobacco as the leading risk factor for cancer DALYs in women before the age of 54.

### Attributable burden of specific cancer types across SDI quintiles

**Figure 5:**
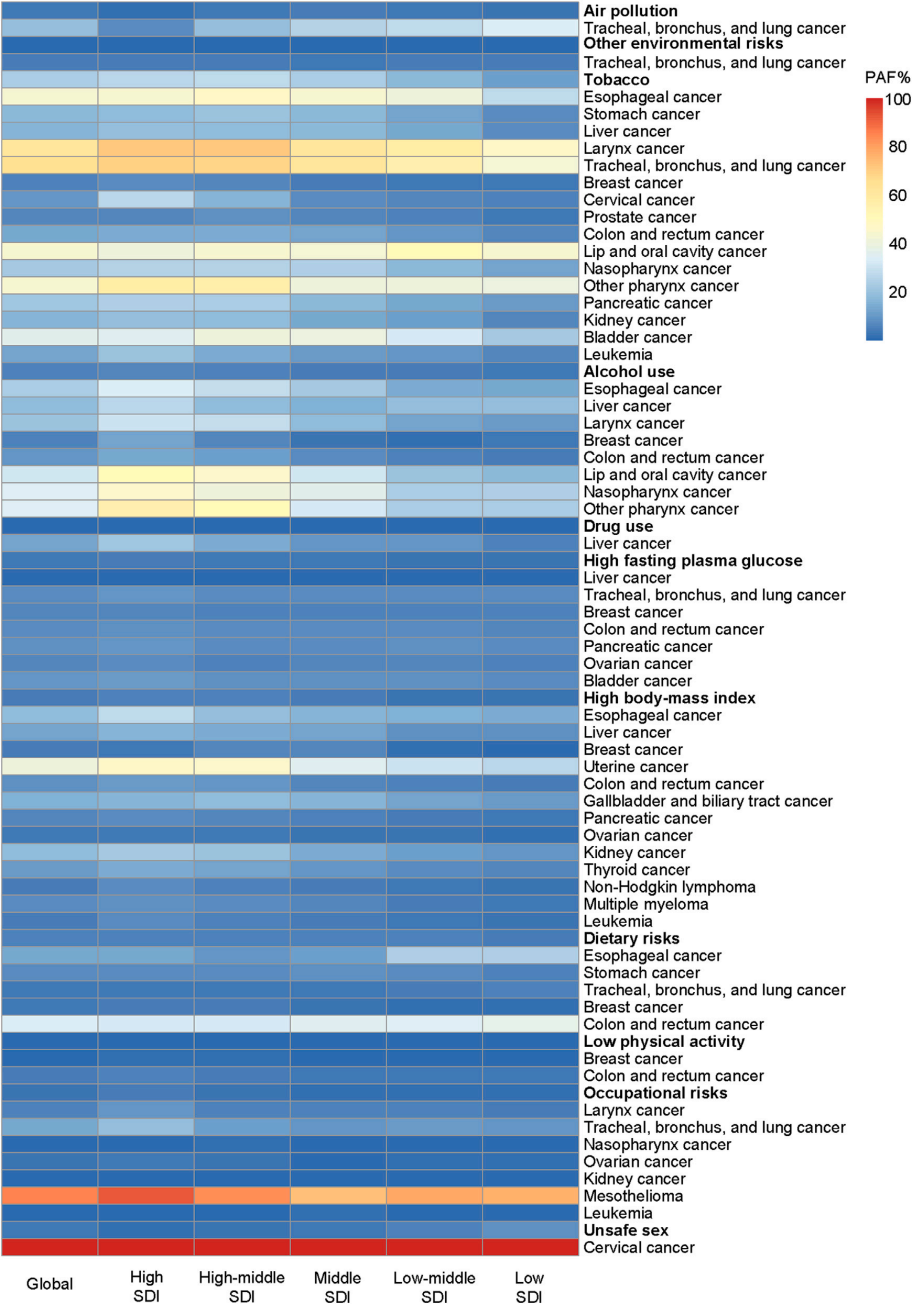
PAFs of cancer DALYs attributable to eleven level 2 risk factors and related cancer types across SDI quintiles in 2019 DALYs, disability-adjusted life years. PAF, population attributable fraction. SDI, sociodemographic index. See also Table S3.

The PAF of type-specific cancer DALYs attributable to risk factors across SDI quintiles is shown in Table S3 and Figure 5. Specifically, tracheal, bronchus, and lung cancer was the only cancer type attributable to air pollution and other environmental risks. Sixteen tumor types were related to tobacco, with the highest PAF found in tracheal, bronchus, and lung cancer at the global level. However, it was worth noteworthy that the PAF for larynx cancer exceeded that for tracheal, bronchus, and lung cancer in high and high-middle SDI quintiles. Alcohol use was associated with 8 categories of tumors, with the highest PAF observed for lip and oral cavity cancer, nasopharynx cancer, and other pharynx cancers. Liver cancer was the only one cancer type associated with drug use. High FPG was correlated with 7 cancer types, with the highest PAF observed in bladder cancer. There were 13 categories of neoplasms associated with high BMI, with the highest PAF observed in uterine cancer. Dietary risks were correlated with five cancer types, with the highest PAF observed in colon and rectum cancer. Low physical activity was associated with breast cancer and colon and rectum cancer. Occupational risks were associated with seven cancers, with the highest PAF observed for mesothelioma. Cervical cancer was associated with unsafe sex, with a PAF of 100% observed for females worldwide.

The PAFs of type-specific cancer DALYs attributable to risk factors were generally higher in higher SDI regions. However, there were exceptions. For example, the PAF attributable to dietary risks differed substantially between SDI quintiles, with the highest PAF observed for esophageal cancer and tracheal, bronchus, and lung cancer in lower SDI quintiles, and for breast cancer in higher SDI quintiles (Table S3; Figure 5).

## Discussion

In 2019, there were 105.0 million cancer DALYs or 41.23% of total cancer DALYs attributable to all level 2 risk factors, with tobacco, dietary risks, alcohol use, high BMI, and air pollution being the top five risk factors. In contrast to the relatively stable trend in PAF for combined risk factors, the PAFs of cancer DALYs attributable to high FPG, high BMI, and low physical activity have been increasing in recent years, due to the economic development and transformation of lifestyle worldwide.

A recent GBD study led by the GBD 2019 Cancer Risk Factors Collaborators reported the cancer deaths and DALYs attributable to potential risk factors.^16^ Because of the same set of data used, most of our findings were comparable to theirs. However, their study only showed the geographical patterns and age distribution of the cancer burden attributable to level 1 risk factors, despite delineation on the sex disparity and SDI patterns of the cancer burden attributable to level 2 risk factors. To raise awareness of these level 2 risk factor estimates, our study highlighted the disparity in sex, age, locations, SDI, and cancer types in terms of cancer burden attributable to level 2 risk factors.

Development status was a significant contributor to cancer burden. The overall PAF was higher in higher SDI regions, suggesting that a higher proportion of cancer burden can be reduced in higher SDI regions if the exposure levels of relevant modifiable risk factors are controlled to the optimal exposure levels. For each specific risk factor, SDI was positively correlated with the PAFs of cancer DALYs attributable to other environmental risks, tobacco, alcohol use, drug use, low physical activity, occupational risks, high FPG and high BMI; negatively correlated with air pollution and unsafe sex; and unrelated to dietary risks. However, development status can only partially explain the variation in PAF across countries, given the complex and non-linear relationship between PAF and SDI. Considerable variations were observed in PAFs among countries, underscoring the need for finer delineation of high-risk populations.

Significant age and sex disparities were observed in the cancer burden attributable to risk factors. Men had a higher PAF attributable to combined risk factors than women, due to a significant contribution from the male-prominent risk factor—tobacco. To be specific, women had higher PAFs attributable to wealth-related risk factors, including high BMI, high FPG, and low physical activity, and unsafe sex than men. Men had higher PAFs attributable to most behavioral, environmental, and occupational risk factors. The highest PAFs were observed in the middle-aged and older adults. Recognizing the long cycle of cancer development, we promoted early prevention to reduce cancer burden. In addition, the sex prominence for a specific risk factor was not consistent in all age groups. For example, men had higher PAF attributable to high BMI before age of 50. However, this pattern was reversed from the 50–54 age group onwards, with a steep increase in the PAF in this age group for women. These findings suggest the need for age- and sex-specific assessments prior to future cancer risk factor interventions.

Tobacco included smoking, secondhand smoke, and chewing tobacco. Tobacco was the leading risk factor in much of the world. Despite a notable decline in tobacco prevalence observed in much of the world, it was still on the rise in many less developed countries, such as Afghanistan, Albania, Saudi Arabia, Lebanon, and Mongolia.^17^ Central Europe and East Asia had the highest cancer death and DALY rates of cancer attributable to tobacco smoking.^13^ As of July 2019, the Tobacco-Free Initiative’s MPOWER policy package has successfully expanded evidence-based tobacco control measures to nearly two-thirds of the world’s population, including raising tobacco taxes, banning tobacco advertisement, and building a smoke-free environment.^18^ In response to this call, every country should spare no effort to accelerate the smoke-free process in the coming decades.

Although alcohol use and tobacco are the two most recognized carcinogens, efforts to reduce alcohol use have been relatively less proactive than for tobacco.^19^ As a result, the PAF of cancer DALYs attributable to alcohol use has increased over the observed periods, especially for men in high-income countries. A related GBD study also found that the cancer burden attributable to alcohol use has decreased from 1990 to 2019, and the attributable burden was higher in males, elders, and developed regions (based on SDI).^15^ Besides, another study reported that an estimated 741 300 or 4.1% of all new cancer cases were attributable to alcohol consumption in 2020, using cancer incidence data from GLOBOCAN 2020.^20^ Because we chose DALYs as the measurement, rather than incidence, the findings of this study were not comparable to ours. Considering a small amount of drinking is likely to be beneficial for cardiovascular function, the World Cancer Research Fund recommends no more than 2 drinks for men and 1 drink for women.^21^

Two forms of air pollution were quantified as contributing to the cancer burden in GBD 2019: ambient particulate matter pollution and household air pollution (HAP) from the use of solid fuels. Despite a large reduction in the prevalence of HAP, the prevalence of ambient particulate matter pollution has increased significantly since the 1990s^22^ The GBD study reported that cancer deaths attributable to ambient particulate matter pollution increased by more than 300% from 1990 to 2017.^23^ Although China has recently experienced reductions in emissions from fossil fuel and solid biofuel, China still had the highest burden from ambient particulate matter pollution.^24^ Given the undesirable situation, the energy, industry, construction, and transport sectors should take action to curb the worsening global air pollution. For example, European legislation on controlling pollution from power generation, manufacturing, and road transport could prevent 163,000 deaths worldwide each year.^25^

Fifteen dietary risk factors were identified by GBD 2019. A relevant study reported that almost 30%–35% of tumors were related to diet.^26^ It was estimated that that for every 50g increase in processed meat intake, the risk of developing cancer increased by 17%. Besides, for every 100g increase in red meat intake, the risk increased by 18%.^27^ On the contrary, regular intake of fresh fruits and vegetables can reduce the risk of stomach cancer, colorectal cancer, and breast cancer.^28^ As more and more countries have adopted the Western dietary style with a large amount of red meat, progressed meat, and insufficient intake of fruits and vegetables, it was in urgent need to construct a healthier diet with easy access to healthy fruits, vegetables, legumes, and whole grains and strict restrictions on the consumption of red meat, processed meat, and sugary drinks worldwide. Several promising interventions include: mass media campaigns, food and menu labeling, food pricing strategies (subsidies and taxes), and dietary legislations et al.^29^

The attributable burden of metabolic risk factors has been increasing worldwide in recent years, particularly high FPG and high BMI. Notably, women were more susceptible to the metabolic risk factors along with low physical activity, because a few metabolic-related cancers such as uterine cancer and breast cancer were prominent in women. A more pronounced gender gap was observed after the age of 54 years, suggesting that these metabolic risk factors pose a greater threat to postmenopausal women than premenopausal women.^30^ The American Cancer Society (ACS) estimates that at least 20% of incident cancers are attributable to obesity, and obese patients had a worse prognosis compared with patients with a normal BMI.^31^ The increasing prevalence of high FPG and high BMI and the determinants behind require further investigation. However, it was universally acknowledged that lack of exercise, excessive intake of calories, undesirable diet quality, and diet composition were closely related to these metabolic risk factors.^32^^,^^33^ A multifactorial means was urgently needed to intervene these metabolic risk factors, such as reducing the intake of sugar, fat, and total calories, and promoting physical activity and exercise to the general public.^34^

Unsafe sex was the leading risk factor in 38 countries and territories, most of which were in Africa. However, the impact of unsafe sex on the cancer burden has not been adequately assessed in previous studies. In our study, unsafe sex was the second leading risk factor responsible for cancer DALYs among women, but surpassed tobacco as the leading risk factor among women before the age of 54. Based on evidence in the literature, GBD 2019 attributed 100% of cervical cancers to unsafe sex. These sources stated that human papillomavirus (HPV) infection, particularly HPV-16 and HPV-18, was necessary for the development of cervical cancer and that HPV was spread only through sexual contact.^35^ In recent years, vaccines against HPV-16 and HPV-18 have been approved for use in adolescents and young women worldwide. However, due to the high cost of developing sufficient vaccines, implementation of vaccines against HPV has not been sufficiently enforced in many less developed countries in sub-Saharan Africa and South Asia.^36^^,^^37^ There is an urgent need to strengthen sexual safety education and expand HPV vaccination among these countries.

Infection was an important un-estimated risk factor in this study, due to the lack of data on the cancer burden attributable to infections in GBD 2019. The PAF of incident cancers attributable to infections was 16% globally in 2008, and higher in low-income countries (22.9%) than in high-income countries (7.4%).^38^ Owing to the poor sanitation and shortage of vaccines, countries in sub-Saharan Africa had the highest PAF (32.7%). Therefore, it is imperative to develop regional frameworks to increase vaccine coverage and improve public health in these target areas in the future.^5^

Studies have shown that prevention strategies targeting modifiable risk factors are cost-effective for cancer prevention. In addition, because of the interaction between risk factors, interference on one risk factor may influence the other one, which could further enhance the effect of risk factor intervention on cancer control.^39^^,^^40^ Epidemiological studies have shown that the effect of individual behavioral intervention is minimal, whereas societal actions such as regulatory controls on risk factors will have a substantial impact on cancer control.^41^ Appropriate approaches include: promoting the significance of primary prevention, adopting and implementing relevant legislations, and further investigating and enforcing the framework for reducing major carcinogens.^42^ Late diagnosis and scarce medical resources remains the major concerns in many less developed countries. Therefore, it is suggested that these countries should strengthen preventive measures along with early screening and palliative care services to reduce cancer burden.^43^

In conclusion, our study provided insight into the magnitude of the cancer burden attributable to 11 level 2 risk factors in 2019 and analyzed the trends over the past decades. We highlighted that a large proportion of cancer DALYs are preventable, with tobacco, dietary risks, alcohol use, high BMI, and air pollution being the top five risk factors. Tobacco remained the leading risk factor in 166 countries and unsafe sex in 38 countries, mainly in Africa. Unsafe sex was considered the female-specific risk factor in GBD 2019, accounting for most cancer DALYs in women before the age of 54. Due to the overwhelming increase in the prevalence, wealth-related risk factors, including high FPG, high BMI, and low physical activity, warranted policy attention worldwide. Our findings may provide insightful information for policymakers to establish cost-effective prevention programs targeting modifiable risk factors to reduce cancer burden in the future.

### Limitations of the study

While there have been significant improvements in data collection and modeling in GBD 2019, the inherent defects of the GBD study should not be ignored. First and foremost, high-quality primary data are scarce in many less-developed countries, where the final estimates must rely on statistical modeling. Even when data are available, the discrepancies in data quality and accuracy can lead to the deviations in the estimates. Secondly, the RRs for each risk factor evaluated may vary across ethnic groups and geographical locations, in which case the use of a universal effect size would reduce the authenticity and reliability of the estimates. Thirdly, prevalence of risk factors and cancer DALYs were obtained in the same year, regardless of the lag time between risk factor exposure and cancer development.^44^ Thus, for a selected risk factor, its estimated PAF may have been overestimated when it was rising and underestimated when it was declining during the observed periods. Lastly, GBD 2019 provided relevant data only when there was solid evidence of a causal relationship between selected risk factors and cancer. Future GBD rounds should explore the attributable cancer burden for other potential risk factors.

## STAR★Methods

### Key resources table

**Table:** 

REAGENT or RESOURCE	SOURCE	IDENTIFIER
**Software and algorithms**		

R 4.0.5	R	http://www.r-project.org/
Figdraw	Figdraw	https://www.figdraw.com/

**Other**		

Source data	Global Health Data Exchange	https://vizhub.healthdata.org/gbd-results/

### Resource availability

#### Lead contact

Further information and requests for resources should be directed to and will be fulfilled by the Lead Contact, Da-Wei Ye (dy0711@gmail.com).

#### Materials availability

This study did not generate new unique reagents.

#### Data and code availability

• All data in this study are publicly accessible (https://vizhub.healthdata.org/gbd-results/).
• This paper does not generate original code. R codes used in this study have been provided in Data S1.
• Any additional information required to reanalyze the data reported in this paper is available from the lead contact upon request.

### Experimental model and study participant details

Participants information on sex, age, and race was self-reported. Information on gender and socioeconomic status was not collected.

### Method details

#### Data sources

The data in our study were obtained from the Global Burden and Disease Study (GBD) 2019 via the online GBD Results Tool: https://vizhub.healthdata.org/gbd-results/. GBD 2019 estimated the disease burden in 2019 and analyzed the temporal trend from 1990 to 2019 for 369 diseases and injuries, and 87 risk factors. In this study, we extracted the attributable DALYs for 11 level 2 risk factors by sex and age from 1990 to 2019 in 204 countries and territories.

The estimation of the disease burden attributable to risk factors followed the comparative risk assessment (CRA) framework, which was based on the premise of how many outcomes can be gained by controlling the exposure level of a given risk factor to the theoretical minimum risk exposure level (TMREL).The general methodology of modelling and estimation has been specified elsewhere.^2^^,^^22^^,^^35^^,^^45^ Here, we provide a snapshot of the data processing in GBD 2019.

#### Risk-outcome pairs

A total of 481 risk-outcome pairs were selected as meeting the evidence grading criteria of being convincing or probable provided by the World Cancer Research Fund.^46^ These risk factors were divided into four hierarchies, with level 1 representing the overarching categories including behavioral, environmental, occupational and metabolic; level 2 included 20 individual risks or risk clusters; level 3 risk factors were the individual risk disaggregated from risk clusters within level 2; level 4 detailed risks in the most disaggregated manner.^22^ The GBD risk hierarchy with levels is presented in Table S4. We extracted 11 level 2 risk factors related to cancer, including air pollution, other environmental risks, tobacco, alcohol use, drug use, high FPG, high BMI, dietary risks, low physical activity, occupational risks, and unsafe sex. Twenty-three cancers were associated with these 11 risk factors. Table S5 shows the International Classification of Diseases (ICD) codes mapped to the cancer mortality data in this study.

#### Exposure estimation

The exposure data come from many sources, including authoritative systematic reviews, large cohort studies, household surveys, national censuses, and satellite data. To make the exposure data from different sources more consistent and suitable for modelling, adjustments were made to the exposure data to deal with alternative case definitions or study methods prior to modelling. Two models, Bayesian meta-regression model (DisMod-MR 2.1) and a spatiotemporal Gaussian process regression model (ST-GPR), were utilized to control and adjust for variation from different sources and to estimate the mean exposure of each risk factor by age, sex, country, and year. The exposure distribution among individuals was modelled by the method of moments (MoM) and then described as mean and standard deviation (SD).^22^^,^^35^

#### Relative risks

The relative risks (RR) for each risk factor come from many sources, including RCTs, cohort, pooled cohort, and case-control studies et al. For high FPG and high BMI, which were estimated from a continuous exposure distribution, the effect size was reported by category in pooled or meta-analysis studies. GBD 2019 converted these categories to RR per unit increase in exposure and assumed a linear increase in the log of the RR and exposure. For a selected set of continuous risk factors, including dietary risk factors, low physical activity, and air pollution, GBD 2019 modelled RRs using meta-regression-Bayesian, regularized, trimmed (MR-BRT), relaxing the log-linear assumption to allow for monotonically increasing or decreasing but non-linear functions using cubic splines.^22^^,^^35^

#### Theoretical minimum-risk exposure level

Theoretical minimum-risk exposure level (TMREL) is the theoretically possible risk exposure level that minimizes the risk to the exposed population. For harmful risk factors with increasing functions, the TMREL was set to 0. For risk factors with an inverted V-shaped function, the value of exposure at the nadir was determined to represent the TMREL.^22^ For protective risks with monotonically decreasing risk functions (e.g., fruit intake), GBD 2019 set the 85th percentile of exposure as the TMREL.

#### Population-attributable fractions

The population attributable fraction (PAF) was calculated as the proportion of disease burden that can be reduced if exposure to a given risk factor is at the TMREL.

When exposure is a continuous variable, the PAF of attributable DALYs for a given risk factor was calculated using the following formula:

$$\text{PAF}=\frac{\int_{x=l}^{m}RR\left(x\right)P\left(x\right)dx-RR\left(x\right)\text{TMREL}}{\int_{x=l}^{m}RR\left(x\right)P\left(x\right)dx}\text{,}$$

where l is the minimum exposure level, m is the maximum exposure level, RR (x) is the relative risks at exposure level x, TMREL is the counterfactual exposure distribution, and P(x) is the current exposure distribution. Each variable is calculated within the incorporation of covariates of age, sex, location, year, and cancer type.

When exposure is a discrete variable, PAF was calculated by this formula:

$$\text{PAF}=\frac{\sum_{x=1}^{m}RR\left(x\right)P\left(x\right)-RR\left(x\right)\text{TMREL}}{\sum_{x=1}^{m}RR\left(x\right)P\left(x\right)}\text{.}$$

Assuming that all risk factors are independent and unrelated to each other, we estimated the PAF of the multiplicative effect of individual risk factor using this formula: PAF = 1-$\prod_{i=1}^{n}(1-{PAF}_{i}$), where i represents an individual risk and n is the number of risk factors.^7^^,^^22^

#### Estimate the attributable burden

DALYs (disability-adjusted life years) were considered the optimal measurement of disease burden, combining YLLs (life years lost due to premature death) and YLDs (years lived with disability weighted for severity by the disability weights). The PAF for each disease cause due to the given risk factor was multiplied by the total DALYs for that cause to estimate the attributable DALYs, by age, sex, location, and year.

#### Sociodemographic index

The socio-demographic index (SDI) is the geometric mean of an index of the total fertility rate of persons under 25 years of age (TFU25), average education of 15 years or more (EDU15+), and the lagging distribution of per capita income,^22^ which is closely related to the health care status of each country or territory. In GBD 2019, each SDI value was multiplied by 100 for a range from 0 (worst) to 100 (best). Based on the SDI values, 204 countries and territories were divided into five quintiles: low SDI, low-middle SDI, middle SDI, high-middle SDI, and high SDI quintiles.

### Quantification and statistical analysis

Age-standardized DALYs rate (ASDR) is used to evaluate and compare the cancer DALY rates among locations with distinct age structures and demographic characteristics. Gaussian process regression with a Loess smoother was fitted to estimate the expected values of PAF within each SDI unit. Spearman rank order correlation methods was utilized to analyze the correlation between PAF and SDI. Statistical significance was determined as a p value less than 0.05. All metrics were estimated with a 95% uncertainty interval (UI). ASDR was reported per 100,000 population. All data processing, analysis, and visualization were performed on the R version 4.2.3.

## Supplemental information

Document S1. Figures S1‒S3 and Table S4

Table S1. Estimates of rates and PAFs of cancer DALYs attributable to eleven level 2 risk factors for both sexes in 2019 in 27 global and regional locations, related to Figure 2 DALYs, disability-adjusted life years. PAF, population attributable fraction.

Table S2. Estimates of PAFs of cancer DALYs attributable to eleven level 2 risk factors for both sexes in 2019 in 204 countries and territories, related to Figure 3 DALYs, disability-adjusted life years. PAF, population attributable fraction.

Table S3. Estimates of rates and PAFs of cancer DALYs attributable to eleven level 2 risk factors for both sexes in 2019, by cancer types and SDI, related to Figure 5 DALYs, disability-adjusted life years. PAF, population attributable fraction. SDI, sociodemographic index.

Table S5. International Classification of Diseases (ICD) codes mapped to the cancer mortality data in this study, related to STAR Methods

Data S1. R codes used in this study, related to STAR Methods

## References

[1] GBD 2019 Diseases and Injuries Collaborators (2020). Global burden of 369 diseases and injuries in 204 countries and territories, 1990-2019: a systematic analysis for the Global Burden of Disease Study 2019. Lancet.

[2] Global Burden of Disease Cancer C., Kocarnik J.M., Compton K., Dean F.E., Fu W., Gaw B.L., Harvey J.D., Henrikson H.J., Lu D., Pennini A. (2021). Cancer Incidence, Mortality, Years of Life Lost, Years Lived With Disability, and Disability-Adjusted Life Years for 29 Cancer Groups From 2010 to 2019: A Systematic Analysis for the Global Burden of Disease Study 2019. JAMA Oncol..

[3] Fitzmaurice C., Dicker D., Pain A., Hamavid H., Moradi-Lakeh M., MacIntyre M.F., Allen C., Hansen G., Woodbrook R., Global Burden of Disease Cancer Collaboration (2015). The Global Burden of Cancer 2013. JAMA Oncol..

[4] Weiderpass E. (2010). Lifestyle and cancer risk. J. Prev. Med. Publ. Health.

[5] Vineis P., Wild C.P. (2014). Global cancer patterns: causes and prevention. Lancet.

[6] Sullivan R., Homberg L., Purushotham A.D. (2012). Cancer risk and prevention in a globalised world: solving the public policy mismatch. Eur. J. Cancer.

[7] Kulhánová I., Znaor A., Shield K.D., Arnold M., Vignat J., Charafeddine M., Fadhil I., Fouad H., Al-Omari A., Al-Zahrani A.S. (2020). Proportion of cancers attributable to major lifestyle and environmental risk factors in the Eastern Mediterranean region. Int. J. Cancer.

[8] Melaku Y.A., Appleton S.L., Gill T.K., Ogbo F.A., Buckley E., Shi Z., Driscoll T., Adams R., Cowie B.C., Fitzmaurice C. (2018). Incidence, prevalence, mortality, disability-adjusted life years and risk factors of cancer in Australia and comparison with OECD countries, 1990-2015: findings from the Global Burden of Disease Study 2015. Cancer Epidemiol..

[9] Chen W., Xia C., Zheng R., Zhou M., Lin C., Zeng H., Zhang S., Wang L., Yang Z., Sun K. (2019). Disparities by province, age, and sex in site-specific cancer burden attributable to 23 potentially modifiable risk factors in China: a comparative risk assessment. Lancet. Glob. Health.

[10] Gapstur S.M., Drope J.M., Jacobs E.J., Teras L.R., McCullough M.L., Douglas C.E., Patel A.V., Wender R.C., Brawley O.W. (2018). A blueprint for the primary prevention of cancer: Targeting established, modifiable risk factors. CA. Cancer J. Clin..

[11] Parkin D.M., Boyd L., Walker L.C. (2011). 16. The fraction of cancer attributable to lifestyle and environmental factors in the UK in 2010. Br. J. Cancer.

[12] Safiri S., Nejadghaderi S.A., Karamzad N., Kaufman J.S., Carson-Chahhoud K., Bragazzi N.L., Sullman M.J.M., Beyranvand M.R., Mansournia M.A., Almasi-Hashiani A. (2022). Global, Regional and National Burden of Cancers Attributable to High Fasting Plasma Glucose in 204 Countries and Territories, 1990-2019. Front. Endocrinol..

[13] Safiri S., Nejadghaderi S.A., Abdollahi M., Carson-Chahhoud K., Kaufman J.S., Bragazzi N.L., Moradi-Lakeh M., Mansournia M.A., Sullman M.J.M., Almasi-Hashiani A. (2022). Global, regional, and national burden of cancers attributable to tobacco smoking in 204 countries and territories, 1990-2019. Cancer Med..

[14] Safiri S., Karamzad N., Kaufman J.S., Nejadghaderi S.A., Bragazzi N.L., Sullman M.J.M., Almasi-Hashiani A., Mansournia M.A., Collins G.S., Kolahi A.A., Jemal A. (2022). Global, regional, and national burden of cancers attributable to excess body weight in 204 countries and territories, 1990 to 2019. Obesity.

[15] Safiri S., Nejadghaderi S.A., Karamzad N., Carson-Chahhoud K., Bragazzi N.L., Sullman M.J.M., Almasi-Hashiani A., Mansournia M.A., Collins G.S., Kaufman J.S., Kolahi A.A. (2022). Global, regional, and national cancer deaths and disability-adjusted life-years (DALYs) attributable to alcohol consumption in 204 countries and territories. Cancer.

[16] (2022). The global burden of cancer attributable to risk factors, 2010–19: a systematic analysis for the Global Burden of Disease Study 2019. Lancet.

[17] GBD 2019 Tobacco Collaborators (2021). Spatial, temporal, and demographic patterns in prevalence of smoking tobacco use and attributable disease burden in 204 countries and territories, 1990-2019: a systematic analysis from the Global Burden of Disease Study 2019. Lancet.

[18] World Health Organization (2019).

[19] Vaish R., Bajpai J. (2021). Alcohol and Cancer: Waiting for the Storm to Pass or Dancing in the Rains. Indian J. Med. Paediatr. Oncol..

[20] Rumgay H., Shield K., Charvat H., Ferrari P., Sornpaisarn B., Obot I., Islami F., Lemmens V.E.P.P., Rehm J., Soerjomataram I. (2021). Global burden of cancer in 2020 attributable to alcohol consumption: a population-based study. Lancet Oncol..

[21] World Cancer Research Fund, American Institute for Cancer Research (2007).

[22] GBD 2019 Risk Factors Collaborators (2020). Global burden of 87 risk factors in 204 countries and territories, 1990-2019: a systematic analysis for the Global Burden of Disease Study 2019. Lancet.

[23] Roth G.J.T.L. (2018).

[24] McDuffie E., Martin R., Yin H., Brauer M. (2021). Global Burden of Disease from Major Air Pollution Sources (GBD MAPS): A Global Approach. Res. Rep. Health Eff. Inst..

[25] Crippa M., Janssens-Maenhout G., Dentener F., Guizzardi D., Sindelarova K., Muntean M., Van Dingenen R., Granier C. (2016). Forty years of improvements in European air quality: regional policy-industry interactions with global impacts. Atmos. Chem. Phys..

[26] Logan J., Bourassa M.W. (2018). The rationale for a role for diet and nutrition in the prevention and treatment of cancer. Eur. J. Cancer Prev..

[27] Key T.J., Bradbury K.E., Perez-Cornago A., Sinha R., Tsilidis K.K., Tsugane S. (2020). Diet, nutrition, and cancer risk: what do we know and what is the way forward?. BMJ.

[28] Wiseman M. (2008). The second World Cancer Research Fund/American Institute for Cancer Research expert report. Food, nutrition, physical activity, and the prevention of cancer: a global perspective. Proc. Nutr. Soc..

[29] World Health Organization (2009).

[30] Rose D.P., Vona-Davis L. (2010). Interaction between menopausal status and obesity in affecting breast cancer risk. Maturitas.

[31] Eyre H., Kahn R., Robertson R.M., ACS/ADA/AHA Collaborative Writing Committee (2004). Preventing cancer, cardiovascular disease, and diabetes: a common agenda for theAmerican Cancer Society, the American Diabetes Association, and the American Heart Association. CA. Cancer J. Clin..

[32] Swinburn B.A., Kraak V.I., Allender S., Atkins V.J., Baker P.I., Bogard J.R., Brinsden H., Calvillo A., De Schutter O., Devarajan R. (2019). The Global Syndemic of Obesity, Undernutrition, and Climate Change: The Lancet Commission report. Lancet.

[33] GBD 2016 Risk Factors Collaborators (2017). Global, regional, and national comparative risk assessment of 84 behavioural, environmental and occupational, and metabolic risks or clusters of risks, 1990-2016: a systematic analysis for the Global Burden of Disease Study 2016. Lancet.

[34] Rezende L.F.M.D., Sá T.H.D., Markozannes G., Rey-López J.P., Lee I.M., Tsilidis K.K., Ioannidis J.P.A., Eluf-Neto J. (2018). Physical activity and cancer: an umbrella review of the literature including 22 major anatomical sites and 770 000 cancer cases. Br. J. Sports Med..

[35] GBD 2019 Cancer Risk Factors Collaborators (2022). The global burden of cancer attributable to risk factors, 2010-19: a systematic analysis for the Global Burden of Disease Study 2019. Lancet.

[36] Sankaranarayanan R. (2009). HPV vaccination: the promise & problems. Indian J. Med. Res..

[37] Hakim A.A., Dinh T.A. (2009). Worldwide impact of the human papillomavirus vaccine. Curr. Treat. Options Oncol..

[38] de Martel C., Ferlay J., Franceschi S., Vignat J., Bray F., Forman D., Plummer M. (2012). Global burden of cancers attributable to infections in 2008: a review and synthetic analysis. Lancet Oncol..

[39] Markussen M.S., Veierød M.B., Kristiansen A.L., Ursin G., Andersen L.F. (2016). Dietary patterns of women aged 50-69 years and associations with nutrient intake, sociodemographic factors and key risk factors for non-communicable diseases. Public Health Nutr..

[40] Ngamwong Y., Tangamornsuksan W., Lohitnavy O., Chaiyakunapruk N., Scholfield C.N., Reisfeld B., Lohitnavy M. (2015). Additive Synergism between Asbestos and Smoking in Lung Cancer Risk: A Systematic Review and Meta-Analysis. PLoS One.

[41] Joshi R., Chow C.K., Raju P.K., Raju K.R., Gottumukkala A.K., Reddy K.S., Macmahon S., Heritier S., Li Q., Dandona R., Neal B. (2012). The Rural Andhra Pradesh Cardiovascular Prevention Study (RAPCAPS): a cluster randomized trial. J. Am. Coll. Cardiol..

[42] Espina C., Porta M., Schüz J., Aguado I.H., Percival R.V., Dora C., Slevin T., Guzman J.R., Meredith T., Landrigan P.J., Neira M. (2013). Environmental and occupational interventions for primary prevention of cancer: a cross-sectorial policy framework. Environ. Health Perspect..

[43] Knaul F.M., Atun R., Bhadelia A. (2012).

[44] Arnold M., Pandeya N., Byrnes G., Renehan P.A.G., Stevens G.A., Ezzati P.M., Ferlay J., Miranda J.J., Romieu I., Dikshit R. (2015). Global burden of cancer attributable to high body-mass index in 2012: a population-based study. Lancet Oncol..

[45] Hu J.J., Dong Y.M., Ding R., Yang J.C., Odkhuu E., Zhang L., Ye D.W. (2023). Health burden of unbalanced fatty acids intake from 1990 to 2019: A global analysis. Med.

[46] Marmot M., Atinmo T., Byers T., Chen J., Hirohata T., Jackson A., James W., Kolonel L., Kumanyika S., Leitzmann C. (2007).

