# Supplementary material for: Cancer burden attributable to risk factors, 1990–2019: A comparative risk assessment
Source: iScience. 2024 Mar 8;27(4):109430. doi: 10.1016/j.isci.2024.109430 (PMC10972825; doi:10.1016/j.isci.2024.109430)
Supplement: Document S1. Figures S1‒S3 and Table S4 [file mmc1.pdf]

iScience, Volume *27*

Supplemental information

Cancer burden attributable to risk factors, 1990–2019: A comparative risk assessment

Junjie Hu, Hongliang Dong, Yiming Dong, Runxuan Zhou, Wilhem Teixeira, Xingxing He, Da-Wei Ye, and Gang Ti

A

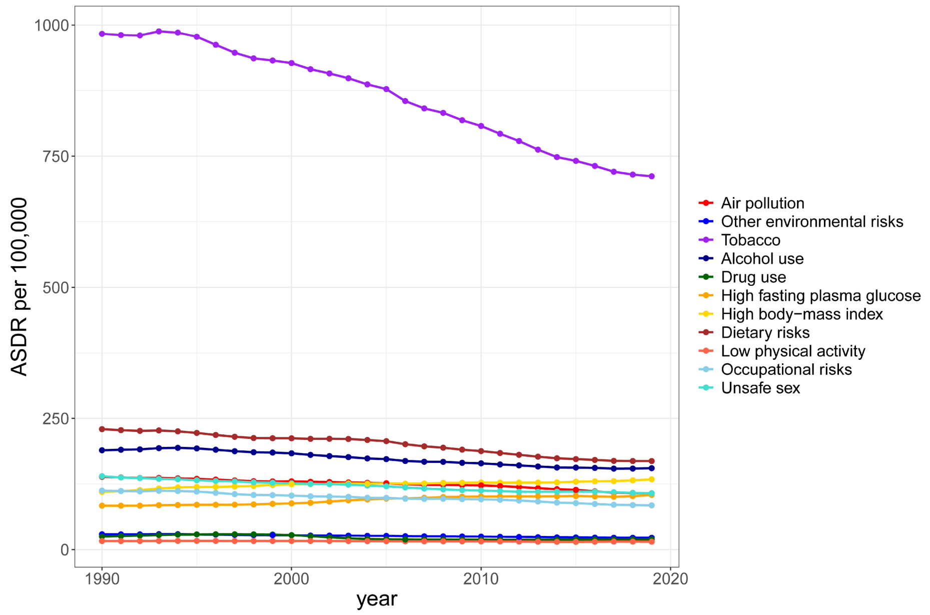

B

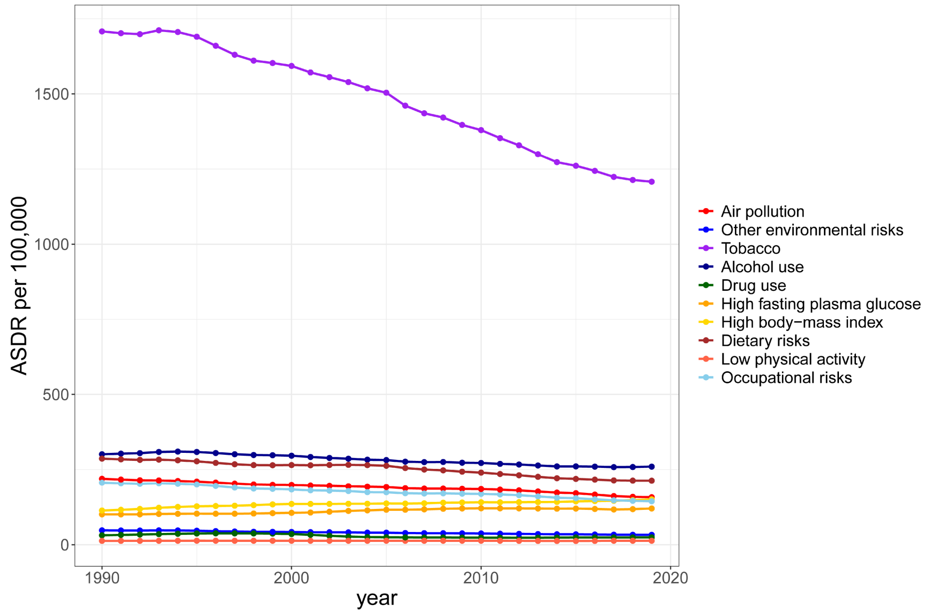

C

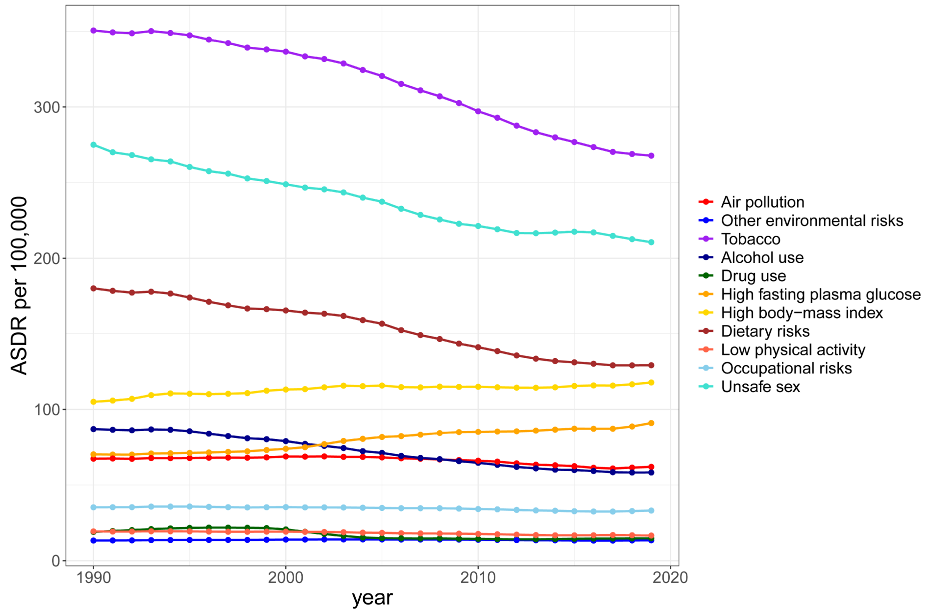

Figure S1. DALY rates of cancer attributable to eleven level 2 risk factors at the global level; related to Figure 1.

The temporal trends of global cancer ASDR per 100,000 people attributable to eleven level 2 risk factors for both sexes (A), male (B) and female (C) from1990 to 2019.

ASDR, age-standardized DALYs rate. DALYs, disability-adjusted life years.

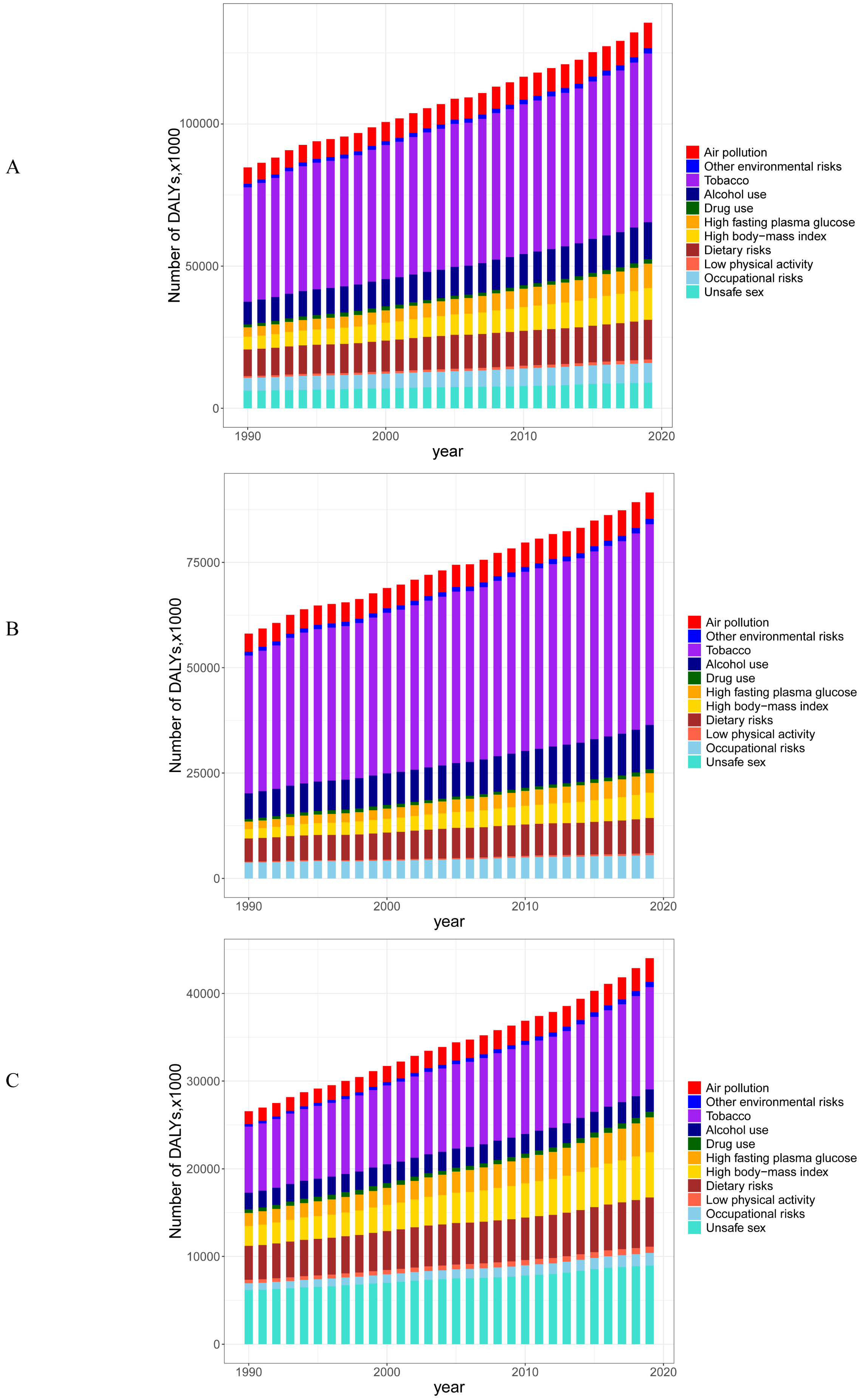

Figure S2. DALYs of cancer attributable to eleven level 2 risk factors at the global level; related to Figure 1.

The temporal trends of global cancer DALYs attributable to eleven level 2 risk factors for both sexes (A), male (B) and female (C) from1990 to 2019.

DALYs, disability-adjusted life years.

Figure S3

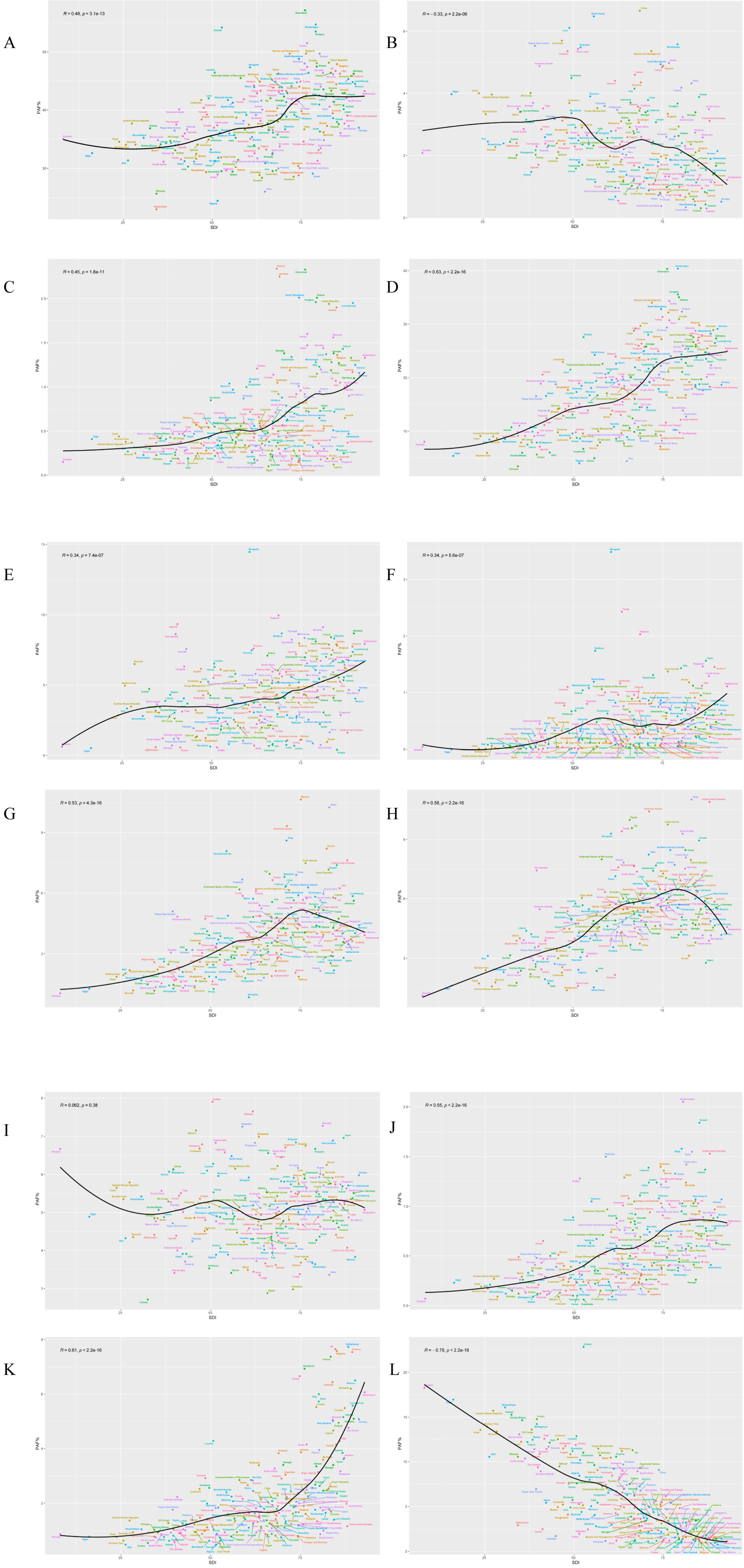

Figure S3. Correlations between PAFs of cancer DALYs attributable to eleven level 2 risk factors and SDI at the national level; related to Figure 3.

The correlations between age-standardized PAF of cancer DALYs attributable to combined modifiable risk factors (A) and eleven specific level 2 risk factors: air pollution (B), other environmental risks (C), tobacco (D), alcohol use (E), drug use (F), high fasting plasma glucose (G), high body-mass index (H), dietary risks (I), low physical activity (J), occupational risks (K), unsafe sex (L), for both sexes and SDI in 204 countries and territories in 2019.

Spearman rank order correlation methods was utilized to analyze the correlation between PAF and SDI. Statistical significance was determined as a p value less than 0.05.

DALYs, disability-adjusted life years. PAF, population attributable fraction. SDI, sociodemographic index.

Table S4. GBD risk hierarchy with levels; related to STAR Methods

Risk	Level
All risk factors	0
Environmental/occupational risks	1
Unsafe water, sanitation, and handwashing	2
Unsafe water source	3
Unsafe sanitation	3
No access to handwashing facility	3
**Air pollution**	**2**
**Particulate matter pollution**	**3**
**Ambient particulate matter pollution**	**4**
**Household air pollution from solid fuels**	**4**
Ambient ozone pollution	3
Non-optimal temperature	2
High temperature	3
Low temperature	3
**Other environmental risks**	**2**
**Residential radon**	**3**
Lead exposure	3
**Occupational risks**	**2**
**Occupational carcinogens**	**3**
**Occupational exposure to asbestos**	**4**
**Occupational exposure to arsenic**	**4**
**Occupational exposure to benzene**	**4**
**Occupational exposure to beryllium**	**4**
**Occupational exposure to cadmium**	**4**
**Occupational exposure to chromium**	**4**
**Occupational exposure to diesel engine exhaust**	**4**
**Occupational exposure to formaldehyde**	**4**
**Occupational exposure to nickel**	**4**
**Occupational exposure to polycyclic aromatic hydrocarbons**	**4**
**Occupational exposure to silica**	**4**
**Occupational exposure to sulfuric acid**	**4**
**Occupational exposure to trichloroethylene**	**4**
Occupational asthmagens	3
Occupational particulate matter, gases, and fumes	3
Occupational noise	3
Occupational injuries	3
Occupational ergonomic factors	3
Behavioral risks	1
Child and maternal malnutrition	2
Suboptimal breastfeeding	3
Non-exclusive breastfeeding	4
Discontinued breastfeeding	4
Child growth failure	3
Child underweight	4
Child wasting	4
Child stunting	4
Low birthweight and short gestation	3
Short gestation	4
Low birthweight	4
Iron deficiency	3

Vitamin A deficiency	3
Zinc deficiency	3
**Tobacco**	**2**
**Smoking**	**3**
**Chewing tobacco**	**3**
**Secondhand smoke**	**3**
**Alcohol use**	**2**
**Drug use**	**2**
**Dietary risks**	**2**
**Diet low in fruits**	**3**
**Diet low in vegetables**	**3**
Diet low in legumes	3
**Diet low in whole grains**	**3**
Diet low in nuts and seeds	3
**Diet low in milk**	**3**
**Diet high in red meat**	**3**
**Diet high in processed meat**	**3**
Diet high in sugar-sweetened beverages	3
**Diet low in fiber**	**3**
**Diet low in calcium**	**3**
Diet low in seafood omega-3 fatty acids	3
Diet low in polyunsaturated fatty acids	3
Diet high in trans fatty acids	3
**Diet high in sodium**	**3**
Intimate partner violence	2
Childhood sexual abuse and bullying	2
Childhood sexual abuse	3
Bullying victimization	3
**Unsafe sex**	**2**
**Low physical activity**	**2**
Metabolic risks	1
**High fasting plasma glucose**	**2**
High LDL cholesterol	2
High systolic blood pressure	2
**High body-mass index**	**2**
Low bone mineral density	2
Kidney dysfunction	2

Note, reference from the GBD 2019 risk hierarchy.[1,2] The risk factors depicted in bold were included in this study.

References

1. Collaborators, G.B.D.R.F. (2020). Global burden of 87 risk factors in 204 countries and territories, 1990-2019: a systematic analysis for the Global Burden of Disease Study 2019. Lancet *396*, 1223-1249. 10.1016/S0140-6736(20)30752-2.
2. Collaborators, G.B.D.C.R.F. (2022). The global burden of cancer attributable to risk factors, 2010-19: a systematic analysis for the Global Burden of Disease Study 2019. Lancet *400*, 563-591. 10.1016/S0140-6736(22)01438-6.
